# Green Extraction Approach for Isolation of Bioactive Compounds in Wild Thyme (*Thymus serpyllum* L.) Herbal Dust—Chemical Profile, Antioxidant and Antimicrobial Activity and Comparison with Conventional Techniques

**DOI:** 10.3390/plants13060897

**Published:** 2024-03-20

**Authors:** Živan Mrkonjić, Muammer Kaplan, Sanja Milošević, Danica Božović, Aleksandra Sknepnek, Dunja Miletić, Ivana Lazarević Mrkonjić, Dušan Rakić, Zoran Zeković, Branimir Pavlić

**Affiliations:** 1 Faculty of Technology, University of Novi Sad, Bulevar Cara Lazara 1, 21000 Novi Sad, Serbia; zivan_mrkonjic@hotmail.com (Ž.M.); sanjamilosevic9898@gmail.com (S.M.); danica_bozovic@live.com (D.B.); ivana.lazarevic702@gmail.com (I.L.M.); dusan.rakic@uns.ac.rs (D.R.); zzekovic@tf.uns.ac.rs (Z.Z.); 2 TUBITAK Marmara Research Centre, Institute of Chemical Technology, P.O. Box 21, 41470 Gebze, Kocaeli, Turkey; 3 Faculty of Agriculture, Institute of Food Technology and Biochemistry, University of Belgrade, 11000 Belgrade, Serbia; aleksandras@agrif.bg.ac.rs (A.S.); dunja.miletic@agrif.bg.ac.rs (D.M.)

**Keywords:** *Thymus serpyllum*, hydrodistillation, Soxhlet extraction, supercritical fluid extraction, essential oil

## Abstract

The aim of this study was to provide a chemical profile and determine the antioxidant and antimicrobial activity of the essential oil (EO) and lipid extracts of *Thymus serpyllum* L. herbal dust obtained via conventional (hydrodistillation (HD) and Soxhlet extraction (SOX)) and novel extraction techniques (supercritical fluid extraction (SFE)). In addition, a comparative analysis of the chemical profiles of the obtained EO and extracts was carried out, as well as the determination of antioxidant, antibacterial and antifungal activity of the lipid extracts. According to the aforementioned antioxidant and antimicrobial activities and the monoterpene yield and selectivity, SFE provided significant advantages compared to the traditional techniques. In addition, SFE extracts could be considered to have great potential in terms of their utilization in the pharmaceutical and cosmetic industries, as well as appropriate replacements for synthetic additives in the food industry.

## 1. Introduction

One of the most taxonomically complex genera of family Lamiaceae, which includes 250–350 species and varieties of wild growing species of herbaceous sub- and perennial shrubs, is genus *Thymus* [1]. It is widely spread across Europe, North Africa and Asia and represents a great potential in terms of its medicinal use through the wide spectrum of its pharmacological properties. *Thymus* species, being considered important medicinal plants, have been used as healing agents in traditional medicine for thousands of years [2]. Different species of *Thymus* possess different types of bioactive compounds that directly affect their chemical compositions and pharmacological activities. In general, the most common compounds identified are thymol and carvacrol, as well as flavonoids and phenolic compounds [3]. One of the well-known medicinal plants and a member of the genus *Thymus* is *Thymus vulgaris* L., which contains more essential oil (EO) than other species and has a great potential as an antioxidant, antimicrobial, anti-inflammatory, antiviral and insecticidal agent [4]. Beside aforementioned, Thymus serpyllum L. represents an aromatic and medicinal plant with a highly potent source of bioactive compounds with antioxidant, antimicrobial, antitumor and cytotoxic properties. Additionally, wild thyme has been used for centuries in traditional medicine, where its most common application was to treat health problems related to respiratory and gastrointestinal systems [5]. It may be used as a substitution for synthetic antioxidants to treat pathological conditions caused by free radicals but also as a substitution for synthetic additives in the food industry for the purpose of reducing food deterioration and extending the product’s shelf-life [6,7]. In order to isolate bioactive compounds from the herb, several conventional and novel extraction techniques have been used. In the first place, conventional hydrodistillation (HD) is considered one of the most convenient for essential oil isolation. Another interesting and widely used technique, which represents a gold standard for the isolation of lipophilic compounds, is Soxhlet extraction (SOX). Although this method is not the most suitable technique in terms of moving production from lab to industrial levels, considering the obtained total extraction yields (Y), it could be used as a great comparative technique. On the other hand, what has been long sought after are novel extraction techniques, advanced in reducing the costs of time, energy, manpower and solvents, as well as increasing the qualitative and quantitative composition of the final product. Last but not least is ensuring a simpler flow of down-stream processes. Precisely in that manner, the novel extraction techniques can respond to the challenges of the modern age to a greater or lesser extent. To extract polyphenolic compounds from *T. serpyllum* L., besides conventional solid–liquid extraction [8,9], also used were extractions assisted with ultrasounds [8,9] and microwaves [10], pulsed electric fields extraction [11], extractions with subcritical fluids [12], as well as extractions with natural deep eutectic solvents (NADESs) [13].

When focusing on the isolation of lipophilic compounds, supercritical fluid extraction (SFE) has to be taken into account as one of the most promising techniques. Although, at the moment, it cannot be seen as economically profitable, its exceptional advantage in obtaining an extract rich in bioactive compounds without traces of solvents places this technique in a very high position in relation to the mentioned techniques. One successful example of herbal dust utilization using SFE was described by Mrkonjić et al. [14], who developed various mathematical models for fitting the *T. serpyllum* SFE process but without insight into chemical profiling and bioactivity. With this in mind, the SFE of *T. serpyllum* herbal dust has been chosen in this work as the technique that is, at the moment, the most suitable for comparison with conventional techniques in terms of the obtained Y, antioxidant and antimicrobial activity and the quantity and quality profile of extracted compounds.

The EOs and lipid extracts consist of a mixture of volatile terpenes, their oxygenated derivatives and also non-volatile compounds that make their separation and identification difficult [15]. Gas chromatography coupled with time-of-flight mass spectrometry (GC-TOF/MS) that is characterized by a high resolution that enables the fast identification of a large number of compounds in EOs was used in order to perform the complete determination of chemical profile of EO and lipid extracts.

Several papers focused on the chemical composition of the EO of *T. serpyllum*, where the presence of secondary metabolites such as terpenes, terpenoids and polyphenolic compounds was determined, have been published [16,17,18,19]. However, to the best of our knowledge, this is the first paper where the GC-TOF/MS of *T. serpyllum* herbal dust SFE extracts has been carried out.

The main objective of our study was to provide the chemical profile of extracts and EO obtained due to conventional and SFE techniques from *T. serpyllum* herbal dust. Additionally, the determination of their Y and antioxidant and antimicrobial activity was conducted in order to evaluate the advantages and disadvantages of hydrodistillation, SFE and Soxhlet extraction.

## 2. Results and Discussion

### 2.1. Total Extraction Yield (Y) and Chemical Composition

**Figure 1:**
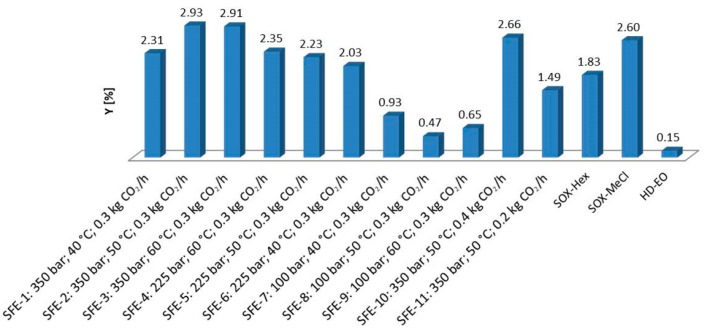
Y of EO, SOX and SFE extracts Antioxidant activity of EO, SOX and SFE extracts determined via DPPH and ABTS methods.

SFE, SOX and HD were applied to determine the Y of the EO and lipid extracts (Figure 1).

The SFE extraction yield ranged from 0.47 to 2.93%, while the Y for SOX-Hex and SOX-MeCl was 1.83 and 2.60%, respectively. Conventional HD gave considerably the lowest yield of wild thyme EO (0.15%). Considering that EO consists of volatile compounds without non-volatile lipids, it could be expected that the EO yield is lower compared to other techniques. Furthermore, according to Y, SFE stood out as the best technique. The highest Y was obtained under the following extraction conditions: pressure of 350 bar, temperature of 50 °C and flow rate of 0.3 kg CO_2_/h. By comparing several different conditions under which the extraction was performed, it can be concluded that the highest yield was achieved by increasing the pressure. Varying the pressure from 100 to 225 bar leads to a sudden increase in Y. Furthermore, the increase in pressure from 225 to 350 bar, enables obtaining the highest Y. Observing the temperature ranged from 40 to 60 °C under the pressure of 350 bar, temperature of 50 °C stood out as the best, which leads to reduced possibility of the thermal degradation of the target compounds. However, a higher flow rate of 0.4 kg CO_2_/h (2.66%) did not enable a higher Y compared to a flow rate of 0.3 kg CO_2_/h (2.93%) at the same pressure and temperature. SOX gave a significantly lower Y of wild thyme EO using hexane as the solvent (1.83%). However, methylene chloride stood out as a better solvent in terms of Y (2.60%). A wide spectrum of compounds characterized by wild thyme EO and extracts were identified via HS-GC-MS (Table 1).

Among the 54 compounds which contribute to the composition of SFE extracts, carvacrol, thymol, borneol, nonane, *o*-cymene, isothymol methyl ether and β-bisabolene were the most widespread. The SFE-11 extract (350 bar, 50 °C, 0.2 kg CO_2_/h) was characterized by the highest content of *o*-cymene (284.66 μg/mL), followed by carvacrol (72.38 μg/mL), isothymol methyl ether (46.74 μg/mL), thymoquinone (43.72 μg/mL), thymol (35.07 μg/mL) and β-bisabolene (32.04 μg/mL). On the other hand, in the SFE-2 extract (350 bar, 50 °C, 0.3 kg CO_2_/h), *m*-cymene (79.34 μg/mL), nonane (26.32 μg/mL), isothymol methyl ether (21.54 μg/mL), carvacrol (20.94 μg/mL), thymoquinone (15.37 μg/mL) and β-bisabolene (15.15 μg/mL) were identified in the highest amounts (Figure S1a). Although SFE extracts emerged as the best in terms of Y, they are not distinguished as the best in terms of terpene content. However, the SFE-10 extract (350 bar, 50 °C, 0.4 kg CO_2_/h) was the most terpene-rich extract. *o*-cymene (167.72 μg/mL), carvacrol (108.15 μg/mL), β-bisabolene (50.32 μg/mL), thymol (49.36 μg/mL) and 5-methyl-2-(1-methylethenyl)-, (R)-4-hexen-1-ol (43.15 μg/mL), were identified in this extract in the highest amounts. Subtle differences in composition between SFE extracts under different conditions could be observed. Although the SFE-2 extract was found to be the best in terms of Y, it was not the richest in terms of terpenes content. The results indicated that extracts obtained at the higher flow, pressure and temperature are richer in terpene content. When SOX was analyzed, the SOX-Hex extract dominantly contained nonane (27.15 μg/mL), carvacrol (19.83 μg/mL), geraniol (13.26 μg/mL) and *o*-cymene (10.68 μg/mL), whose chromatogram could be found in Figure S1b. The SOX-Hex extract was richer in monoterpenes content compared to SOX-MeCl. Although a higher yield of lipophilic extracts was obtained with methylene chloride, it does not represent an adequate solvent in terms of terpene content. Even though they were obtained via different techniques, SFE and SOX extracts do not differ significantly in terms of the content of dominant compounds. The most significant results were obtained via HD. Wild thyme EO was characterized by a significantly higher content of *m*-cymene (832.26 μg/mL), γ-terpinene (192.52 μg/mL), α-terpinyl acetate (177.69 μg/mL) and isothymol methyl ether (151.73 μg/mL). α-copaene, β-thujene, β-myrcene, β-cububene, 2-hexenal, benzaldehyde, *cis*-sabinenehydrate, dihydrocarvone, hexanal and α-terpinyl acetate were identified only in HD-EO (Figure S1c). Considerable differences can be observed by comparing HD extracts with SOX and SFE extracts, mostly due to the absence of non-volatile lipids in HD-EOs. Therefore, HD represents the most economically profitable technique for obtaining oils with a high content of volatile terpenes. However, in terms of the environmental aspects and extraction yield, the advantage is given to SFE.

Sfaei-Ghomi et al. [20] proved that there are small differences in the composition of EOs obtained via HD between four different *Thymus* species (*T. persicus*, *T. eriocalyx*, *T. daenensis* subsp. *daenensis* and *T. serpyllum* L.). Carvacrol (14.94%), α-pinene (12.2%) and thymol (7.39%) were the major terpenes in *T. serpyllum*, with an EO yield of 1.2 ± 0.8%, which was in accordance with the present data. Furthermore, similar results were reported from *T. serpyllum* EO from the Mascara region [6]. The obtained EO yield rich in carvacrol (66%), γ-terpinene (11.5%), thymol (7.5%) and *p*-cymene (3.9%) was 5.66%, which is higher than the Y obtained in this work. Bendif et al. [21] performed SFE, pressurized liquid extraction (PLE) and HD to obtain the extraction yield, chemical composition and antioxidant activity of the two *Thymus munbyanus* subspecies [21]. SFE extracts (45 MPa, 70 °C and 2 L CO_2_/min) achieved a Y of 0.35 to 0.43%, which is significantly less than the results obtained by the same technique in this work. The main reasons for this are the extraction parameters, which could have led to the precipitation of waxes in the separator. However, the yield of EO obtained via HD (0.11 and 0.09%) was similar to the Y obtained in this work (0.15%). Comparing *Thymus* species collected from different regions of Iran, it was detected that thymol (12.4–79.74%), carvacrol (4.37–42.14%), geraniol (0.3–22.44%) and *p*-cymene (0.8–12.86%) were the most dominant [22]. *T. migricus* provides a higher Y (3.87%) in comparison with *T. fedtschenkoi*-2 (0.29%). These yields are higher than the Y obtained in this work, so it can be concluded that differences in species are noticeable. Pavlić et al. [23] used sage herbal dust for discovering the adequate technique in terms of Y among HD, SOX and SFE. SOX stood out with both methylene chloride and hexane (14.68 and 10.84%, respectively), which is not in accordance with the results obtained in this work. Also, wild thyme is characterized by a significantly lower yield compared to sage. The differences in the chemical composition of the EO and extracts between these two plants are notable, even though they belong to the same plant family. Camphor, α-thujone, eucalyptol, viridiflorol and epirosmanol were the most abundant compounds in sage herbal dust, opposite to the wild thyme, where thymol, carvacrol and *o*-cymene were the most dominant.

Also, Pavlić et al. [24] applied HD, SOX, microwave-assisted extraction (MAE), ultrasound-assisted extraction (UAE) and SFE in order to obtain peppermint EO and lipophilic extracts. According to the GC-MS results, the most dominant compounds in peppermint extracts were monoterpenes. Among these monoterpenes, the most abundant compounds were menthol, menthone, isomenthol, isomenthone and eucalyptol. They concluded that the different techniques applied have a huge impact on the extracts’ chemical profile because of their different selectivity. Kulisic et al. [25] proved that there is no particular difference in the qualitative composition between thyme and wild thyme EOs obtained via HD. GC-MS analysis showed that the predominant compounds in both thyme and wild thyme were γ-terpinene, *p*-cymene, thymol and carvacrol, which is in correlation with the results in this study. Another study from Aćimović el al. [17] examined the chemical composition of Mediterranean plants such as *S. kitaibelii*, *T. serpyllum* and *O. vulgare*, which belong to the Lamiaceae family. The obtained results showed that the most abundant compounds in *T. serpyllum* EO were geraniol (63.4%) and nerol (18.9%), which is not in accordance with the results obtained in this work. In addition, *S. kitaibelii* EO contained *p*-cymene, limonene and linalool, while *O. vulgare* EO contained germacrene D, 1,8-cineole, sabinene and *trans*-caryophyllene. Differences in the composition of terpenes between the same species, but also between species which belong to the same family, can be caused by growing conditions, geographical location and environmental factors. An investigation by Goyal et al. [19] confirmed variation in the dominant compounds (thymol, α-terpineol, *p*-cymene, camphor and γ-terpinene) in *T. serpyllum*. They observed that the content of thymol was different in three locations, Haldwani (84.63%), Auli (50.80%) and Pithoragarh (41.15%). In addition to thymol, which was dominant, camphor was the second most abundant terpene (36.34%). According to Verma et al. [26], *T. serpyllum* showed an extraction yield of 0.22%, whereas, using GC-MS, it was determined that the most abundant compounds were thymol, *p*-cymene, thymol methyl ether, borneol, sabinene, γ-terpinene and carvacrol methyl ether, which is in accordance with EO composition obtained in this work. In addition, Topal et al. [27] determined the chemical composition of nine EOs from different Turkish plants. Among them, *T. serpyllum* was used to obtain steam distillation (SD) EO and SFE extracts. GC-MS analysis showed that the dominant terpenes in SFE extracts were *p*-cymene (5.41%), carvacrol (47.79%), 2,4,6-trimethylanisole (24.95%) and β-bisabolene (3.67%). The content of thymol in SFE extracts and in SD EO was 1.53 and 1.41%, respectively. The results obtained in these studies are in accordance with the presented results in this paper, and both show that thymol was one of the most dominant compounds found in the extracts.

In order to identify the complete composition of wild thyme EO and SFE extracts, the sophisticated GC-TOF/MS method was used (Table 2).

SFE-2, SFE-7 and HD-EO were selected accordingly. SFE-2 (350 bar; 50 °C; 0.3 kg CO_2_/h) was selected on the basis of the obtained Y, as well as because of the milder possibility of thermal degradation in the comparison with SFE-3 (350 bar; 60 °C; 0.3 kg CO_2_/h) (Figure 1). Low values of pressure and temperature proved to be suitable for the extraction of cyclic and acyclic oxygenated monoterpenes, while for the extraction of aromatic oxygenated monoterpenes, conditions of elevated pressure and temperature are used [28]. Therefore, the sample SFE-7 (100 bar; 40 °C; 0.3 kg CO_2_/h) was selected. According to potentially the richest chemical composition and the possibility of being used as a good example for comparative analysis with SFE extracts, the HD-EO sample was selected. The most abundant compounds in the SFE-2 extract (350 bar, 50 °C, 0.3 kg CO_2_/h) were fatty acids *cis*-vaccenic acid (10.445%) and n-hexadecanoic acid (0.413%), pentacosane (0.728%), octacosane (0.704%), monoterpenes and their derivatives, carvacrol (2.058%), geraniol (0.468%), 2-methoxy-1-methyl-4-(1-methylethyl)-benzene (0.380%), endo-borneol (0.298%) and triterpene squalene (0.474%). The chemical composition of the SFE-7 extract was more complex when compared to SFE-2. In comparison with SFE-2, where 151 different compounds were identified, in SFE-7 (100 bar, 40 °C, 0.3 kg CO_2_/h), a total of 199 compounds were identified. It can be concluded that the number of identified compounds increases under milder conditions of SFE extraction; in other words, potential nonselectivity could be noticed compared to SFE-2 conditions. The main identified compounds were fatty acids *trans*-13-octadecenoic (8.888%), *cis*-vaccenic (4.269%) and n-hexadecanoic acid (0.786%), pentacosane (0.696%), octacosane (0.924%), monoterpenes and their derivatives, phenol, carvacrol (1.317%), geraniol (0.572%), *p*-cymene (0.736%), limonene (0.318%), 2-methoxy-1-methyl-4-(1-methylethyl)-benzene (0.656%), endo-borneol (0.561%) and triterpene squalene (0.813%). There are many similarities in the chemical composition of SFE extracts obtained under different process parameters. Among the monoterpenes, carvacrol was present in both extracts as the main compound. The presence of 242 compounds was identified in the sample HD-EO, which represents the richest sample in terms of chemical composition. The main terpenoid compounds were neral (0.803%), β-phellandrene (0.538%), β-bisabolene (0.563%), *p*-cymene (0.534%), geraniol (1.687%), limonene (0.638%), carvacrol acetate (1.231%), 2-methoxy-1-methyl-4-(1-methylethyl)-benzene (0.726%), α-terpinene (0.695%), α-terpinyl propionate (0.921%), (E)-3,7-dimethyl-2,6-octadienal (0.814%), bornyl acetate (0.526%), endo-borneol (0.978%) and δ-cadinene (0.406%). In addition to terpenes, phthalic acid, cyclobutyl tridecyl ester (0.789%), oleic (4.312%) and *cis*-vaccenic acid (0.377%) were the most abundant compounds in the wild thyme EO. Geraniol (1.687%) and carvacrol acetate (1.231%) stood out as the dominant terpenes in EO, which is different compared to the SFE extracts composition. Variations in chemical composition are the cause of using different extraction techniques and process parameters. Even though the EO gave the lowest Y, it is characterized by a very diverse chemical composition and a high content of terpenoid compounds. Monoterpenes and oxygenated monoterpenes stood out as the most abundant class of terpenes, with minor differences in amounts among all extracts.

Although there are no studies dealing with a GC-TOF/MS analysis of *T. serpyllum* so far, there are several papers on the topic of the chemical composition of Lamiaceae family plants. Among them, Shashiashvili el al. [29] investigated the EO composition from *Perilla nankinensis* via the GC-TOF/MS method. Moreover, 28 compounds were identified in EO, and among them, the most abundant were (Z,E)-(α-farnesene)3,7,11-trimethyl-1,3,6,10-dodecatetraene (34.3%), caryophyllene oxide (10.2%), linalool (10.2%) and humulene (3.9%). Ozel et al. [30] compared the composition of different EOs from *Origanum onites* by using comprehensive GC×GC-TOF/MS. Subcritical water extraction (SWE), SD and SOX were applied to obtain EOs. The authors concluded that varying the temperature could also cause a change in EO composition. A similar trend can be noticed between SFE extracts obtained in different conditions in this research. Similar to this work, IIi et al. [31] identified, using two-dimensional GC-TOF/MS, carvacrol, borneol, terpinen-4-ol, 2-caren-10-al, linalool, (*Z*)-*α*-terpineol, thymol and *o*-cymene as the most dominant compounds in *O. onites* EOs. SD and SOX were performed for obtaining EOs as well. Among the 32 compounds from *O. onites*, carvacrol (59.71 and 62.06%) was identified as the most dominant, which is in correlation with the presented data in this work. Carvacrol was obtained as the most abundant in *Thymbra spicata* leaves as well (74.47 and 77.15%). Kutlular et al. [32] used superheated water extraction for obtaining EOs from the leaves and grains of *O. onites*. Among 40 compounds, carvacrol (84.83%) was the main compound detected via GC-TOF/MS, which is similar to *T. serpyllum* EO. On the other hand, β-pinene (0.93%), *p*-cymene (0.61%), linalool (5.14%), borneol (0.79%), terpinen-4-ol (0.86%), α-terpineol (0.56%) and thymol (1.45%) were also present in *O. onites* EOs.

### 2.2. In Vitro Antioxidant Activity

**Figure 2:**
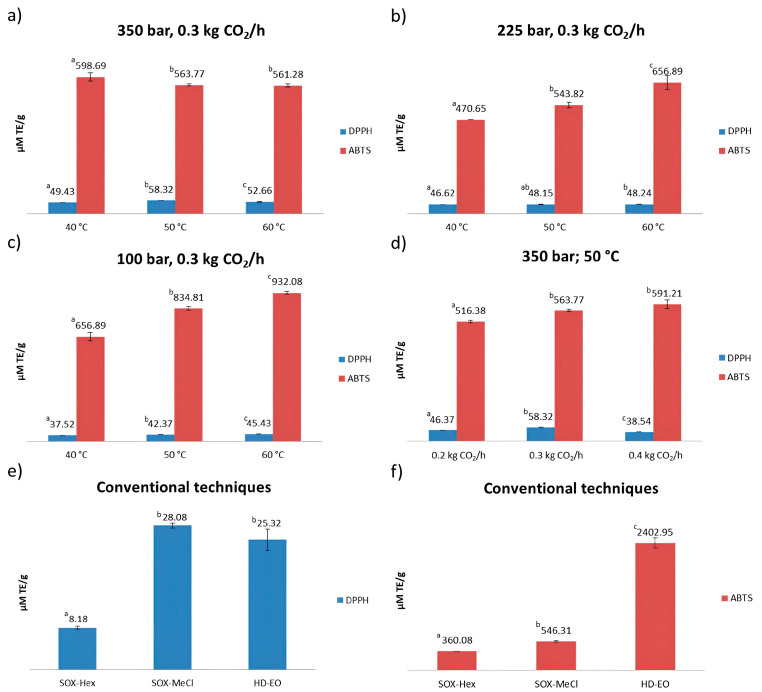
Antioxidant activity of (**a**) SFE extracts obtained at 350 bar and 0.3 kg CO_2_/h by varying the temperature, (**b**) SFE extracts obtained at 225 bar and 0.3 kg CO_2_/h by varying the temperature, (**c**) SFE extracts obtained at 100 bar and 0.3 kg CO_2_/h by varying the temperature, (**d**) SFE extracts obtained at 350 bar and 50 °C by varying the CO_2_ flow rate, (**e**) SOX extracts and HD-EO determined via DPPH and (**f**) SOX extracts and HD-EO determined via ABTS method. Means values obtained via DPPH and ABTS assays marked by different letters are significantly different at α = 0.05 (Tukey’s HSD).

The ability to neutralize DPPH and ABTS^+^ radicals was determined in samples obtained via SFE, SOX and HD. The results of the antioxidant capacity of wild thyme extracts determined by DPPH and ABTS methods are presented in Figure 2.

The DPPH test values ranged from 8.18 to 58.32 µM TE/g, where the highest antiradical scavenging effect was shown in extracts obtained via SFE at the temperatures of 50 and 60 °C and at a fixed pressure (350 bar) and CO_2_ flow rate (0.3 kg CO_2_/h) (58.32 µM TE/g and 52.66 µM TE/g, respectively) (Figure 2a). Regarding the DPPH assay, the sample SOX-Hex showed the lowest antioxidant activity (8.18 µM TE/g) (Figure 2e). In addition, at the pressure of 225 bar and with a CO_2_ flow rate of 0.3 kg CO_2_/h, using different temperatures (40, 50 and 60 °C), it was observed that there were no big changes in antioxidant activity (Figure 2b). However, in the case of the ABTS assay, by increasing the temperature, the possibility of neutralizing ABTS^+^ radicals increased as well (Figure 2b). In order to visualize the impact of the CO_2_ flow rate on the antioxidant activity of SFE extracts, a pressure of 350 bar and temperature of 50 °C were used (Figure 2d). It could be concluded that in the case of DPPH, there is no notable effect of the CO_2_ flow rate, in comparison with ABTS, where antioxidant activity increased with the elevated CO_2_ flow rate. According to the DPPH values of samples obtained via conventional techniques, the sample SOX-MeCl showed the highest antioxidant activity (28.08 µM TE/g), followed by HD-EO (25.32 µM TE/g), while SOX-Hex showed the lowest antioxidant activity (8.18 µM TE/g) (Figure 2e). In the case of ABTS, the sample HD-EO (2402.95 µM TE/g) showed four to six times higher antioxidant activity compared to the samples obtained via SOX (Figure 2f). It could be concluded that the pressure had the greatest influence on antioxidant activity in the case of the DPPH test, where by increasing the pressure, antioxidant activity increases as well. An increase in the pressure could cause the increase in CO_2_ density, which directly affects the increase in its solvation power. Considering a close relationship between the solubility of target compounds and the solvent density, greater antioxidant activity and also the low selectivity of the extraction process toward the targeted compounds and the low level of purity of the obtained extracts could have occurred [33].

Pavlić et al. [23] determined in vitro antioxidant activity from sage herbal dust extracts obtained using SFE, HD and SOX. A high DPPH value of SFE extracts was obtained at a pressure of 300 bar, temperature of 50 °C and CO_2_ flow rate of 0.3 kg CO_2_/h (987.60 mM TE/g). These results were obtained under nearly identical conditions as in this paper. However, the sage herbal dust extract showed higher antioxidant effect than the wild thyme extract. Kulisic et al. [25] determined the antioxidant activity of thyme and wild thyme EOs that were obtained via HD. According to the DPPH assay, EO thyme had a slightly better antioxidant activity compared to wild thyme. Babovic et al. [34] isolated an antioxidant fraction from thyme via SFE at a pressure of 35 MPa, temperature of 100 °C and CO_2_ flow rate of 0.3 kg CO_2_/h. The results of the DPPH test were presented as the IC_50_ value, where the radical scavenging of the thyme extract was 0.08 mg/mL. This result was compared with synthetic antioxidant butylated hydroxytoluene (BHT), whose value of IC_50_ was slightly better (0.03 mg/mL). Topal et al. [27] analyzed samples obtained via SD and SFE. They used nine Turkish plants from different families, including *T. serpyllum* from the Lamiaceae family. In this study, the EO of *T. serpyllum* showed effective DPPH radical scavenging potential when compared with BHT. SFE was performed at a pressure of 20–30 MPa and a temperature of 40–60 °C. The samples obtained via SFE had marginally higher free radical scavenging activity percentages than the EO samples obtained via SD. Similar research was executed by Petrović et al. [35], where bioactive compounds were isolated from *Thymus praecox* using the SFE method under the following conditions: at 100 bar and 40 °C and at 300 bar and 40 °C. At a lower pressure, IC_50_ was 446.0 mg/mL, and with increasing pressure, IC_50_ decreased (404.5 mg/mL), which means that the pressure had a positive impact on the antioxidant activity of the extracts, which corresponds to the results in this work.

The potential to scavenge ABTS^+^ radicals is significantly higher compared to DPPH. ABTS test values ranged from 360.08 to 2402.95 µM TE/g. According to the ABTS test, the highest antioxidant activity was shown by HD-EO, while the SOX-Hex and SOX-MeCl showed a notable lower radical scavenging value (360.08 and 546.31 µM TE/g, respectively). Extracts obtained by SFE at a pressure of 100 bar, at a temperature of 50 and 60 °C and at a CO_2_ flow rate of 0.3 kg CO_2_/h (Figure 2c) showed the highest antioxidant activity (834.81 and 932.08 µM TE/g, respectively). The lowest ABTS radical scavenger was shown by the extract obtained by SFE under the following conditions: pressure 225 bar, temperature 40 °C and CO_2_ flow rate of 0.3 kg CO_2_/h (470.65 µM TE/g). When the temperature increased (Figure 2a), at constant pressure (350 bar) and at constant CO_2_ flow rate (0.3 kg CO_2_/h), there was a decrease in antioxidant activity. This can be explained as a consequence of the degradation of antioxidants.

Pavlić et al. [24] evaluated the antioxidant activity of peppermint. According to the DPPH test, sample SOX-MeCl had the highest antioxidant activity (98.43 ± 2.39 mM TE/g). Meanwhile, the ABTS test showed the highest antioxidant activity in the sample obtained via MAE (138.62 ± 4.33 mM TE/g). In the case of SFE, 400 bar, 40 °C and a CO_2_ flow rate were shown to be the best conditions for the obtained extracts with the highest DPPH and ABTS radical scavenging (33.27 ± 3.13 and 47.50 ± 4.02 mM TE/g, respectively). Compared to the results obtained in this work, it could be concluded that peppermint has a greater ability to reduce DPPH and ABTS^+^ radicals than wild thyme. Similar results are reported in the review paper by Šojić et al. [7], who have found a practical application of *T. serpyllum* SFE extracts obtained at the following conditions: pressure of 100 bar, temperature of 40 °C for the first extract, and pressure of 350 bar and temperature of 50 °C for the second extract. In both extractions, the flow rate was 0.3 kg CO_2_/h. Leon-Méndez et al. [36] also reported that *T. vulgaris* EO, obtained via HD, showed good antioxidant activity obtained by the DPPH assay (IC_50_ = 165.5 ± 1.05 μg/mL), while the IC_50_ value obtained by the ABTS assay was 29.07 ± 0.07 μg/mL. In addition, the antioxidant activity of *T. vulgaris* EO obtained via HD was reported by Gladikostić et al. [37] as well. The value of the DPPH assay was 29.78 µM TE/g, which is very similar to the results in this work. In the case of the ABTS assay, the antioxidant activity value was 757.19 µM TE/g, which is significantly lower compared to the results in this paper.

### 2.3. Antimicrobial Activity

The results of antimicrobial activity assay for extracts prepared from *T. serpyllum* by-products are presented in Table 3.

Two Gram-positive bacterial strains, *S. aureus* ATCC 25923 and methicillin-resistant *S. aureus* (MRSA), were the most sensitive to all three samples, with MIC values below 0.02 mg/mL. EO and SFE-2 expressed the same inhibitory effect against *B. spizizeni* (MIC value of 0.31 mg/mL) and, compared to SFE-7 (MIC value of 0.83 mg/mL), were over 2.5 times more effective. Both *E. faecalis* strains and *L. monocytogenes* were the most susceptible to HD-EO samples (Table 3). HD-EO and SFE-2 showed microbicidal effects on all tested Gram-positive bacteria. On the other hand, the MBC of SFE-7 for both *E. faecalis* strains was not determined. *B. spizizeni* (MBC value 0.31 mg/mL for HD-EO and SFE-2) and *S. aureus* ATCC 25923 (MBC value 0.31 mg/mL, SFE-2) stood out as the most sensitive. All three samples had an microbicidal effect on methicillin-resistant *S. aureus* (MBC value of 0.62 mg/mL HD-EO; MBC value of 2.5 mg/mL for SFE-2 and SFE-7). *S. aureus* is a producer of staphylococcal enterotoxin in foods which causes poisoning after ingestion and is a causer of skin infections as well as other systemic infections [38]. Furthermore, the *S. aureus* (MRSA) strain is recognized as a top priority of worldwide public health systems as its prevalence is between 25 and 50%, and in some areas, it reaches over 60% of all *S. aureus* isolates [39]. The outstanding activity of all *T. serpyllum* SFE extracts against *S. aureus* ATCC 25923 and *S. aureus* MRSA fulfills the enormous importance of tackling these problems.

Regarding the Gram-negative bacteria, observed differences between the samples were more pronounced. Namely, sample HD-EO expressed the highest efficiency, with MICs and MBCs established for all bacteria (MIC was between 0.10 and 2.5 mg/mL, and MBC ranged between 0.62 and 2.5 mg/mL). The most sensitive were *P. hauseri* and *Y. enterocolitica* (MIC 0.16 and 0.10 mg/mL, respectively). Additionally, HD-EO was the only sample that had both microbistatic and microbicidal effects on *E. coli* strains (Table 3). Regarding SFE-2 and SFE-7 extracts, differences in antibacterial activity were also observed. Namely, better activity was found for the SFE-2 extract which acted in an inhibitory way toward seven strains in comparison with the SFE-7 sample that inhibited the growth of four Gram-negative strains. *P. hauseri* and *Y. enterocolitica* were the most sensitive strains on both samples, but the MIC values were lower for SFE-2 (0.83 mg/mL) than for SFE-7 (2.5 mg/mL). Comparing MBCs, differences were even more pronounced since SFE-7 had an microbicidal effect only on three bacterial species, i.e., *P. mirabilis*, *P. hauseri* and *P. aeruginosa*, expressing MBC activity that was significantly lower compared with the SFE-2 extract.

Additionally, when comparing SFE-2 and SFE-7, a microbicidal effect on *S.* Typhimurium, *S.* Enteritidis and *Y. enterocolitica* bacterial species was found only for SFE-2. These findings are in accordance with the previous research when the SFE-2 extract, applied in ground pork meat, more efficiently reduced the total *Enterobacteriaceae* number and total number of microorganisms, showing better activity on its microbiological profile than the other extracts. Such an effect was the consequence of the extraction parameters that were set to obtain the total lipid amount and high polyphenolic terpenoids in the SFE-2 extract. The identification of sixteen compounds in SFE-2 that were not found in SFE-7 proved the more complex composition of SFE-2 [7]. Differences between the samples were observed regarding antifungal activity as well. The SFE-7 sample did not express activity on *C. albicans* pathogenic yeast growth. HD-EO and SFE-2 exhibited both fungistatic and fungicidal effects. It can be seen that HD-EO had a fungistatic effect on *C. albicans* yeast at a lower concentration (1.25 mg/mL) compared with SFE-2 (2.5 mg/mL). A similar pattern was observed regarding fungicidal activity, with the MFC eight times lower for HD-EO (1.25 mg/mL) compared to SFE-2 (10 mg/mL). In the research of Jovanović et al. [40], lyophilized *T. serpyllum* extracts obtained after maceration, heat or ultrasound-assisted extractions did not express fungicidal activity against *C. albicans*, while the MIC value of SFE-2 (2.5 mg/mL) was lower. In addition, SFE-2 expressed lower MICs against *S. aureus* and *Y. enterocolitica* and a lower MBC on *S. aureus*, but higher concentrations of SFE-2 were necessary to inhibit the growth of *L. monocytogenes*, *E. coli* and *Salmonella* sp. compared to the lyophilized extracts [40]. Activity absence toward *C. albicans*, *Salmonella* spp., *E. faecalis*, *Ps. aeruginosa*, *Shigella flexneri* and *S. aureus* was determined for hydroalcoholic extracts of T. *serpyllum* [41]. Monoterpene alcohols are usually recognized as components with high antimicrobial activity [38,42]. However, in this research, thymol and carvacol concentrations were lower in the SFE-2 sample (Table 1) that exhibited stronger antibacterial and antifungal activity than the SFE-7 extract. The highest concentration of linalool and terpinen-4-ol were found in HD-EO, followed by SFE-2 and SFE-7, which is in accordance with the detected antimicrobial activity of the samples (Table 3). Similarly, monoterpene hydrocarbons, i.e., camphene, γ-terpinene, *m*-cymene, α-pinene and β-pinene, were found in the highest concentration in HD-EO than in SFE-2 and were not detected in SFE-7 or were present in lower concentrations. These compounds were previously shown to express anticandidal and antibacterial activity [38]. It was previously found that minor components might also contribute to better antibacterial activity via synergistic effects with major components [42,43]. Even though the extracts obtained from *T. serpyllum* by-products acted at higher concentrations in comparison to applied antibiotics (Table 3), natural extracts are attracting high interest for application in food systems to prevent the occurrence of pathogenic microorganisms and to reach a prolonged product shelf-life. The complex chemical composition of phytopreparations that contain several active compounds allows the treatment of microbial infections due to the reduced possibility of resistance development in microorganisms. Additionally, it is possible to reduce the applied doses of antibiotics via the synergistic effects of active compounds from plant extracts with antibiotics [44]. In that manner, the better activity of SFE-2 recommends it for applications in food, cosmeceutical or pharmaceutical products with antimicrobial properties.

## 3. Materials and Methods

### 3.1. Sample

The sample of wild thyme herbal dust was kindly donated by Macval Tea D.O.O. (Novi Sad, Serbia), and it constitutes industrial waste generated during filter tea production. By cutting, grinding and fractionating the raw plant material, a certain amount is separated and considered a by-product because of its mean particle size (≤0.315 mm) and the impracticality of it being packed in the filter tea bags. The *T. serpyllum* herbal dust was stored in paper bags at room temperature prior to the extractions and distillations.

### 3.2. Chemicals

(±)-6-hydroxy-2,5,7,8-tetramethylchromane-2-carboxylic acid (Trolox) and 2,2-diphenyl-1-picrylhydrazyl (DPPH) were supplied from Sigma-Aldrich (Steinheim, Germany). 2,2′-azino-bis(3-ethylbenzothiazoline-6-sulfonic acid) diammonium salt (ABTS) (98%) was purchased from J&K, Scientific Ltd., Beijing, China. Potassium peroxydisulfate was purchased from Lach-Ner, Neratovice, Czech Republic, while sodium acetate anhydrous was purchased from Kemika, Zagreb, Croatia. Carbon dioxide (99.9%) was supplied from Messer Technogas A.D., Novi Sad, Serbia, and the ultra-pure water was obtained via a Milli-Q Plus system (EMD Millipore, Billerica, MA, USA). All other chemicals used were of analytical reagent grade.

### 3.3. Supercritical Fluid Extraction (SFE)

SFE was performed using a high-pressure extraction system (HPEP, NOVA, Swiss, Efferikon, Switzerland), which consists of a gas cylinder with CO_2_, a diaphragm-type compressor (pressure range up to 1000 bar), an extractor with an internal volume of 200 mL (maximal operating pressure of 700 bar), a separator (maximal operating pressure of 250 bar), a temperature regulation system and pressure control and regulation valves. The sample (35.00 g) was placed in the extractor vessel, and the extraction process was carried out by varying the pressure, temperature and CO_2_ flow rate. The separator conditions were fixed at 15 bar and 25 °C, and the extraction time was 180 min. The first 9 extracts were obtained by varying the pressure (100, 225 and 350 bar) and temperature (40, 50 and 60 °C)), while the other SFE extracts were obtained by varying the CO_2_ flow rate (0.2, 0.3 and 0.4 kg CO_2_/h) under a fixed pressure of 350 bar and at a temperature of 50 °C. The Y was measured after a 180 min extraction time and presented as a mass of total extractable solids per 100 g of dry plant material (*w*/*w*). The obtained extracts were collected into plastic vials and stored at 4 °C prior to analysis.

### 3.4. Soxhlet Extraction (SOX)

SOX was performed using a Soxhlet apparatus where wild thyme herbal dust (10.00 g) was extracted with two organic solvents, hexane and methylene chloride (120 mL each), separately. After 6 h, the solvent was evaporated under vacuum, and the obtained extract was further dried at 40 °C for 24 h in a laboratory dryer (Sutjeska, Belgrade, Serbia). The obtained extracts (SOX-Hex and SOX-MeCl) were collected into glass vials and stored at 4 °C prior to analysis.

### 3.5. Hydrodistillation (HD)

HD was performed according to the official HD procedure [45]. The Y was presented as percentage (%, *v*/*w*), and the obtained EO was collected into glass vials and stored at 4 °C prior to analysis.

### 3.6. Analysis of Chemical Composition via GC-MS Techniques

#### 3.6.1. HS-GC-MS

The quantification of identified compounds of *T. serpyllum* extracts and essential oil was achieved using an Agilent gas chromatograph–mass spectrometer equipped with a headspace sampler (HS-GC-MS). The system consists of an Agilent 7697A headspace sampler, a 6890 N gas chromatograph (Santa Clara, CA, USA) and a 5975C mass selective detector (Santa Clara, CA, USA). The chromatographic separation of compounds was performed on a DB-5MS (60 m × 0.25 mm ID, 0.25 m) capillary column. After achieving full equilibrium at 60 °C for 30 min, 1 mL of the headspace sample was injected into a capillary column in split mode (15:1) for 30 s. The transfer line was set at 150 °C. Helium with a constant flow rate at 1 mL/min was used as the carrier gas. Initially, the oven temperature was held at 50 °C for 2 min and then programmed to 300 °C at a rate of 10 °C/min and finally kept at 300 °C for 5 min. The mass spectrometer was operated in the electron ionization mode (70 eV) in the mass scan range of m/z 40–550 Da. The ion source temperature was set at 300 °C. The retention indices (RIs) of compounds were calculated using a series of *n*-alkanes (C_10_–C_24_).

#### 3.6.2. GC×GC-TOF/MS

The identification of volatile compounds of *T. serpyllum* extracts were performed using a LECO Pegasus 4D GC×GC-TOF/MS instrument (LECO Corporation, St. Joseph, MI, USA) equipped with an Agilent 7890B gas chromatograph. A set of non-polar and a mid-polar columns was used for GC×GC separation. The first dimension (1D) column was 30 m × 250 µm × 0.1 µm DB-5 MS (Agilent J&W GC Columns, USA), and the second dimension (2D) column was 2 m × 150 µm × 0.15 µm Rxi-17Sil MS Restek (Bellefonte, PA, USA). GC inlet and transfer line temperatures were set at 220 and 250 °C, respectively. Helium was used as the carrier gas at a constant flow of 1 mL min^−1^. Cryogenic modulation was performed with a 4 s modulation period (PM). Sample injection volumes of 1 mL and split ratios of 10:1 were performed using a CombiPAL autosampler (CTC Analytics, Zwingen, Switzerland). The oven temperature for the first column was held at 40 °C for 1 min and then ramped up to 260 °C at a rate of 5 °C/min and held for 1 min. The second oven was operated at 10 °C higher than the first oven throughout the process. The modulation period was 4 s with a heat pulse of 1 s. A Pegasus^®^ IV time-of-flight mass spectrometer (LECO Corp.) was used as the detector. MS was operated in electron impact ionization mode (70 eV), and ions were collected in the mass range of 45–550 amu. The ion source temperature was set at 230 °C. The tentative identification of compounds was based on a similarity comparison of standard MS in NIST05 (National Institute of Standards and Technology, Gaithersburg, MD, USA) and Wiley (Wiley, New York, NY, USA) libraries.

### 3.7. Antioxidant Activity

The antioxidant activity of the samples was determined via spectrophotometric methods for the scavenging of DPPH [46] and ABTS^+^ radicals [47]. The DPPH assay was performed by mixing 100 μL of the extract with 2900 μL of DPPH solution, which was previously prepared in a concentration of 26 mg/L of methanol and adjusted with the aim of reaching an absorbance of 0.70 ± 0.02 at a wavelength of 517 nm. After the extracts were left for 1 h in the dark and at room temperature, the absorbances were recorded at the same wavelength.

In order to perform the ABTS assay, the ABTS stock was first prepared. Moreover, 7 mM ABTS and 2.45 mM potassium peroxydisulfate were mixed (1:1, *v*/*v*) and stored in the dark at room temperature for 16 h. After 16 h, in order to prepare the ABTS reagent, the ABTS stock was mixed with acetate buffer pH 3.6 (1:40, *v*/*v*). In order to complete the preparation of the ABTS reagent, it was adjusted in order to reach an absorbance of 0.70 ± 0.02 at a wavelength of 734 nm. Subsequently, the ABTS assay could be performed by mixing 100 µL of the extract with 2900 µL of the ABTS reagent. After 5 h in the dark at room temperature, the absorbances were recorded at a wavelength of 734 nm.

All experiments were performed in triplicate, and the mean values were presented as mM of the Trolox equivalent (TE) per gram of the sample dry weight (mM TE/g).

### 3.8. Antimicrobial Activity

#### 3.8.1. Bacterial Strain and Culture Conditions

To determinate the antibacterial activity of the samples, six Gram-positive bacterial strains (*Enterococcus faecalis* ATCC 29212, *Enterococcus faecalis* clinical isolate, *Bacillus spizizeni* ATCC 6633, *Staphylococcus aureus* ATCC 25923, methicillin–resistant *Staphylococcus aureus* MRSA clinical isolate and *Listeria monocytogenes* ATCC 19111) and nine Gram-negative (*Proteus mirabilis* ATCC 12453, *Proteus hauseri* ATCC 13315, *Pseudomonas aeruginosa* clinical isolate, *Escherichia coli* ATCC 25922, *Escherichia coli* H7:O157 ATCC 35150, *Salmonella* ser. Enteritidis ATCC 13076, *Salmonella* ser. Typhimurium ATCC 14028, *Shigella sonnei* ATCC 29930, *Yersinia enterocolitica* ATCC 27729) were used. Antifungal activity of the samples was determined against one pathogenic yeast, *Candida albicans* ATCC 1231. For culturing *E. coli* H7:O157 and *L. monocytogenes* bacteria, tryptic soy agar/broths (HiMedia, Lab., LLC, Mumbai, India) were used, while the other bacteria were cultured in Müller Hinton agar/broth (HiMedia). For the antifungal activity assay, *C. albicans* yeast growth was performed using Malt agar/broth (HiMedia). From the appropriate agar plate, a 24 h old colony was sub-cultured to an adequate broth (5 mL), which was incubated at 37 °C for 18–24 h. The final concentration of microorganisms was adjusted to ~10^5^ colony forming units per mL (CFU/mL) using the DEN-1 McFarland densitometer (Biosan, Riga, Latvia).

#### 3.8.2. Broth Microdilution Method

To determinate the minimal inhibitory (MIC, mg/mL), minimal bactericidal (MBC, mg/mL) and minimal fungicidal concentration (MFC, mg/mL) of the three *T. serpyllum* extracts, the broth microdilution method was used [40,48]. Prior to the testing, the samples were dissolved in 5% DMSO/water solution followed by vigorous stirring. Three-fold sample dilutions in the concentration range between 0.02 mg/mL and 20 mg/mL were prepared in a 96-well microtiter plate (Sarstedt, Nümbrecht, Germany). Bacterial and yeast suspensions were added to each well that contained a sample of the different dilution so that the final volume in each well was 100 µL. After seeding, microtiter plates were incubated at 37 °C for 18–24 h. As a positive control, only bacterial suspensions without the sample were used, while as a negative control, 5% DMSO/water solution was applied. Additionally, to assess the inhibitory activity of the tested samples, chloramphenicol and nystatin were used for bacteria and yeast, respectively. As an indicator of microbial cell growth, resazurin sodium salt was added, and its color change from blue to pink or colorless indicated microbial growth. The lowest concentration of a sample where no visible microbial growth was observed (i.e., there was no change in the indicator color) was designated as the MIC (mg/mL). To determinate the MBC and MFC, sample dilutions with an already established MIC were sub-cultured to the appropriate agar base, and the lowest tested concentration with no visible growth after incubation was designated as the MBC or MFC. The antimicrobial activity assay was performed in triplicate for each sample/microorganism, and the results were represented as the mean value ± standard deviation. The obtained results were analyzed using a single-factor analysis of variance (ANOVA) by means of the statistical program Origin Pro 9.0. The significance of the differences between the samples that were determined were tested using Tukey’s HSD test at a significance level of *p* ≤ 0.05.

## 4. Conclusions

Volatile compounds quantification in wild thyme SFE extracts via GC-MS provided chemical profiles that contained carvacrol, thymol, nonane, *o*-cymene, borneol, isothymol methyl ether and β-bisabolene as the most dominant compounds, while HD-EO was characterized as the terpene-richest sample.

The complete chemical composition of SFE extracts and EO was determined via GC-TOF/MS, where the most different compounds were identified in the sample HD-EO (242) compared to the samples SFE-2 (151) and SFE-7 (199).

The highest Y was observed in SFE extracts, which leads to the conclusion that SFE has an advantage in terms of monoterpene yield and improved selectivity compared to SOX and HD.

According to the antioxidant and antimicrobial activity observed in lipid extracts obtained via SFE, it could be concluded that further research should be aimed at its utilization in the pharmaceutical and cosmetic industries, as well as in the food industry, in order to improve the sensory properties and to secure the prolonged shelf-life of products.

## Figures and Tables

**Table 1:** The quantification of volatile compounds detected in wild thyme EO and SFE and SOX extracts determined via HS-GC-MS.

Compound	RT [min]	RI_exp −_ RI_lit_	SFE-1	SFE-2	SFE-3	SFE-4	SFE-5	SFE-6	SFE-7	SFE-8	SFE-9	SFE-10	SFE-11	SOX-Hex	SOX-MeCl	HD-EO
			[µg/mL]													
(-)-(Z)-β-caryophyllene	26.141	1408	ND	ND	ND	7.92	4.32	4.22	4.97	ND	ND	ND	ND	ND	ND	0.65
(-)-isocaryophyllene	26.819	1408	ND	ND	ND	ND	ND	ND	ND	ND	ND	1.28	0.95	ND	ND	2.95
1-octen-3-ol	16.169	979	ND	ND	ND	ND	ND	ND	ND	10.96	ND	ND	ND	ND	ND	ND
2,2-dimethoxybutane	9.566		ND	ND	ND	ND	1.91	ND	ND	ND	ND	ND	ND	ND	ND	ND
2-methyl-5-(1-methylethyl)-2,5-cyclohexadiene-1,4-dione/thymoquinone	22.785		16.45	15.37	15.06	18.05	7.48	7.43	12.10	31.75	16.13	ND	43.72	ND	6.87	ND
3,7-dimethyl-, (E)-2,6-octadien-1-ol	22.678	1254	8.06	ND	3.29	12.21	3.96	3.02	7.74	ND	ND	ND	ND	ND	7.23	ND
(E)-3,7-dimethyl-2,6-octadien-1-ol, formate/geraniol (59%)	22.736		ND	ND	ND	ND	ND	ND	ND	ND	ND	ND	ND	13.26	ND	ND
2-hexenal	12.586	855	ND	ND	ND	ND	ND	ND	ND	ND	ND	ND	ND	ND	ND	3.59
3-octanol	16.672	988	4.51	4.25	3.85	2.18	1.57	1.43	2.00	12.73	3.25	11.14	13.77	ND	ND	34.67
(R)-5-methyl-2-(1-methylethenyl)-4-hexen-1-ol	22.661		ND	ND	ND	ND	ND	ND	ND	ND	5.37	43.15	ND	ND	ND	ND
Benzaldehyde	15.883	969–960	ND	ND	ND	ND	ND	ND	ND	ND	ND	ND	ND	ND	ND	1.32
Borneol	21.231	1165	6.41	5.97	6.03	7.74	2.72	2.76	5.89	19.68	6.26	18.81	16.71	1.95	2.65	30.98
Camphene	15.562	954	1.92	3.42	2.43	ND	1.03	1.19	ND	1.28	3.28	8.75	14.57	ND	ND	85.47
Camphor	20.636	1141	ND	ND	ND	ND	ND	ND	ND	ND	ND	1.53	2.02	ND	ND	ND
Carvacrol	23.76	1298	23.99	20.94	28.29	76.03	14.55	17.99	61.03	35.71	34.16	108.15	72.38	19.83	21.02	87.35
Caryophyllene oxide	29.464	1583	ND	ND	ND	ND	ND	ND	ND	ND	ND	ND	ND	ND	0.82	0.88
*cis*-linalool oxide	18.687	1067	ND	ND	ND	ND	ND	ND	ND	ND	ND	1.37	1.11	ND	ND	ND
*cis*-sabinenehydrate	18.71	1070	ND	ND	ND	ND	ND	ND	ND	ND	ND	ND	ND	ND	ND	7.48
Dihydrocarvone	21.909	1191/1200	ND	ND	ND	ND	ND	ND	ND	ND	ND	ND	ND	ND	ND	1.22
Eucalyptol	17.76	1035–1031	ND	ND	ND	ND	ND	ND	ND	ND	ND	ND	5.63	ND	ND	19.91
Geraniol 90%	23.066	1249	ND	ND	ND	0.80	ND	ND	ND	ND	ND	ND	ND	ND	ND	2.84
Germacrene D	14.795	930	ND	ND	ND	ND	ND	ND	ND	ND	ND	5.93	9.68	ND	ND	57.21
Hexanal	10.914	801	ND	ND	ND	ND	ND	ND	ND	ND	ND	ND	ND	ND	ND	2.73
Isothymol methyl ether 90%	22.504	1244	16.32	21.54	17.97	9.60	6.65	6.45	8.14	37.58	17.28	38.54	46.74	3.40	5.94	151.73
Limonene	17.657	1035–1029	ND	6.44	8.11	6.95	5.99	5.51	7.59	ND	9.56	ND	ND	ND	ND	ND
Linalool	19.322	1096	7.28	5.74	4.95	4.22	2.46	3.11	4.83	16.59	5.80	21.74	23.36	1.36	1.27	38.87
Linalool acetate 91%	22.694	1257	ND	7.52	ND	ND	ND	ND	ND	20.83	ND	ND	26.32	ND	ND	43.36
*m*-Cymene	17.554	1023	ND	79.34	58.01	ND	ND	ND	15.96	ND	ND	ND	ND	ND	8.58	832.26
Naphthalene-d8 (I.S.)	21.521		2.00	2.00	2.00	2.00	2.00	2.00	2.00	2.00	2.00	2.00	2.00	2.00	2.00	2.00
Neryl acetate	25.215	1359	4.09	3.43	3.20	5.23	1.65	2.22	3.40	6.73	4.89	9.82	10.28	1.91	1.35	15.70
Nonane	13.959	900–900	25.16	26.32	25.03	28.21	27.57	20.66	24.03	ND	25.27	28.58	22.74	27.15	18.90	14.76
*o*-cymene	17.543	1021	53.16	ND	ND	14.40	24.41	25.18	ND	76.62	74.17	167.72	284.66	10.68	ND	ND
Terpinen-4-ol	21.372	1174	4.37	3.83	4.01	4.90	1.34	1.41	ND	ND	4.05	11.16	10.66	ND	ND	14.45
Thymol	23.554	1290	13.79	9.60	11.95	28.97	6.73	8.97	26.17	18.29	15.50	49.36	35.07	8.31	8.30	43.11
Thymol methyl ether 91%	22.273	1232	5.23	6.08	4.85	2.92	2.10	2.14	2.80	10.19	5.04	13.05	16.04	ND	1.83	44.05
α-copaene	25.529	1376	ND	ND	ND	ND	ND	ND	ND	ND	ND	ND	ND	ND	ND	1.26
α-humulene	27.158	1454	ND	ND	ND	ND	ND	ND	ND	ND	ND	1.26	ND	ND	ND	3.05
α-phellandrene	17.05	1010–1002	ND	ND	ND	ND	ND	ND	ND	ND	ND	ND	1.47	ND	ND	9.30
α-pinene	15.047	939	1.93	3.00	1.93	ND	ND	0.89	ND	ND	2.50	7.33	11.67	ND	ND	76.23
α-terpinene	17.314	1018–1017	ND	1.52	ND	ND	ND	ND	ND	0.96	ND	ND	4.52	ND	ND	48.36
α-terpineol	21.669	1188	7.88	5.80	6.08	9.94	3.86	4.67	12.27	17.85	6.75	31.81	22.38	4.75	5.42	28.56
α-terpinolene	19.082	1088	1.17	1.06	1.19	1.13	0.86	0.84	1.19	2.48	1.50	2.94	3.45	ND	ND	18.94
α-terpinyl acetate	24.785	1349	ND	ND	ND	ND	ND	ND	ND	ND	ND	ND	ND	ND	ND	177.69
β-bisabolene	27.844	1505	12.35	15.15	15.82	20.31	9.22	10.91	20.61	16.35	29.41	50.32	32.04	5.91	7.69	58.50
β-bourbonene	25.736	1387	ND	0.79	0.76	ND	ND	ND	ND	1.02	0.83	2.42	1.58	ND	ND	4.29
β-caryophyllene	26.463	1419	7.11	10.90	9.61	ND	ND	ND	ND	12.19	10.61	20.35	16.16	2.09	1.43	59.60
β-cubebene	26.612	1387	ND	ND	ND	ND	ND	ND	ND	ND	ND	ND	ND	ND	ND	1.33
β-myrcene	16.478	988	ND	ND	ND	ND	ND	ND	ND	ND	1.35	ND	ND	ND	ND	37.41
β-pinene	16.295	979	8.26	9.26	6.68	ND	ND	2.75	ND	ND	ND	22.20	33.57	ND	ND	91.06
β-thujene	15.322	966	ND	ND	ND	ND	ND	ND	ND	ND	ND	ND	ND	ND	ND	3.62
γ-cadinene	27.389	1491	ND	ND	ND	ND	ND	ND	ND	ND	ND	1.77	1.06	ND	ND	3.16
γ-terpinene	18.367	1059	2.50	4.38	2.90	ND	1.34	1.23	ND	2.62	4.13	4.15	12.72	ND	1.24	192.52
δ-cadinene	28.141	1523	0.76	1.00	1.07	1.42	ND	0.73	1.30	1.13	0.96	3.60	1.87	ND	ND	5.38

RT—retention time; RI_exp_—Kovat’s retention index calculated; R_Ilit_—retention index reported in the literature; MS—comparison with mass spectra library; ND—not detected.

**Table 2:** The chemical composition of SFE extracts and EO determined via GC-TOF/MS.

Compound	SFE-2	SFE-7	HD-EO
	Relative Percentage (%)		
(-)-β-bourbonene	0.021	0.049	0.341
(1-methylpropyl)-benzene	ND	0.003	ND
(1R)-2,6,6-trimethylbicyclo [3.1.1]hept-2-ene	ND	ND	0.007
(1R,2S,6S,7S,8S)-8-isopropyl-1-methyl-3-methylenetricyclo[4.4.0.02,7]decane-rel-	0.004	ND	ND
(1S,4S,4aS)-1-isopropyl-4,7-dimethyl-1,2,3,4,4a,5-hexahydronaphthalene	ND	ND	0.064
(1S-*cis*)-1,2,3,5,6,8a-hexahydro-4,7-dimethyl-1-(1-methylethyl)-naphthalene/δ-cadinene	ND	ND	0.406
(2α,4aα,8aα)-3,4,4a,5,6,8a-hexahydro-2,5,5,8a-tetramethyl-2H-1-benzopyran	ND	ND	0.059
(3S,3aS,6R,7R,9aS)-1,1,7-trimethyldecahydro-3a,7-methanocyclopenta[8]annulene-3,6-diol	0.004	ND	ND
(3β)-9,19-cyclolanost-24-en-3-ol	0.033	ND	ND
(3β)-olean-12-en-3-ol, acetate	ND	0.018	ND
**(9Z,12Z)-(E)-3,7-dimethylocta-2,6-dien-1-yl octadeca-9,12-dienoate**	0.053	0.110	0.012
(9Z,12Z,15Z)-(E)-3,7-dimethylocta-2,6-dien-1-yl octadeca-9,12,15-trienoate	0.085	0.093	0.009
(All-E)-(±)-2,6,10,15,19,23-hexamethyl-1,6,10,14,18,22-tetracosahexaen-3-ol	0.022	0.095	ND
(All-E)-2,2-dimethyl-3-(3,7,12,16,20-pentamethyl-3,7,11,15,19-heneicosapentaenyl)-oxirane	ND	0.020	ND
(E)-1-(2,6,6-trimethyl-1,3-cyclohexadien-1-yl)-2-buten-1-one	ND	ND	0.068
(E)-1-phenyl-1-butene	ND	0.003	ND
(E)-2,6-dimethylocta-3,7-diene-2,6-diol	0.008	ND	ND
(E)-3,7,11-trimethyl-1,6,10-dodecatrien-3-ol	0.007	ND	ND
(E)-3,7-dimethyl-2,6-octadienal	ND	ND	0.814
(E)-3,7-dimethylocta-2,6-dien-1-yl dodecanoate	ND	0.007	0.005
(E)-3,7-dimethylocta-2,6-dien-1-yl palmitate	0.047	0.013	0.052
(E)-3,7-dimethylocta-2,6-dien-1-yl stearate	ND	0.013	0.011
(E)-3,7-dimethylocta-2,6-dien-1-yl tetradecanoate	ND	0.006	ND
(E)-3-eicosene	ND	ND	0.001
(E)-cinnamaldehyde	ND	ND	0.003
(E,E)-2,4-decadienal	0.007	ND	ND
(E,E)-2,4-heptadienal	0.016	0.021	0.198
(E,E)-2,4-hexadienal	ND	ND	0.016
(E,E)-2,6-dimethyl-2,4,6-octatriene	ND	0.005	ND
(E,E)-3,5-octadien-2-one	ND	0.002	0.097
(E,E)-3,7,11,15-tetramethyl-1,6,10,14-hexadecatetraen-3-ol	0.024	0.030	0.053
(E,E)-6,10,14-trimethyl-5,9,13-pentadecatrien-2-one	ND	ND	0.005
(E,Z)-2,6-dimethyl-2,4,6-octatriene	ND	ND	0.094
(R)-2(4H)-5,6,7,7a-tetrahydro-4,4,7a-trimethyl-benzofuranone	0.046	0.070	0.039
(R)-2-methyl-5-(6-methylhepta-1,5-dien-2-yl)cyclohex-2-enone	0.006	ND	0.110
(R)-4-methyl-1-(1-methylethyl)-3-cyclohexen-1-ol	ND	0.092	ND
(R)-α,α,4-trimethyl-3-cyclohexene-1-methanol/α-terpinyl propionate	ND	ND	0.921
(S,1Z,6Z)-8-isopropyl-1-methyl-5-methylenecyclodeca-1,6-diene	ND	ND	0.047
(S,E)-4-hydroxy-3,5,5-trimethyl-4-(3-oxobut-1-en-1-yl)cyclohex-2-enone	0.004	ND	ND
(Z)-11-hexadecen-1-ol	ND	ND	0.130
(Z)-13-docosenamide	0.007	ND	0.002
(Z)-13-octadecenal	0.025	ND	0.009
(Z)-2-(hexa-2,4-diyn-1-ylidene)-1,6-dioxaspiro[4.4]non-3-ene	ND	ND	0.001
(Z)-3,7-dimethyl-1,3,6-octatriene	0.027	ND	ND
(Z)-3,7-dimethyl-2,6-octadien-1-ol	ND	ND	0.185
(Z)-3,7-dimethyl-2,6-octadien-1-ol formate	ND	0.025	ND
(Z)-3,7-dimethylocta-2,6-dien-1-yl palmitate	0.004	ND	ND
(Z)-9-octadecenal	ND	0.007	ND
(Z)-benzoate, 3-hexen-1-ol	ND	ND	0.034
(Z,Z)-12-octadecadienoic acid, methyl ester	ND	ND	0.009
(Z,Z)-3,6-nonadienal	0.004	0.004	ND
[1S-(1α,4aβ,8aα)]-1,2,4a,5,8,8a-hexahydro-4,7-dimethyl-1-(1-methylethyl)-, [1S-(1α,4aβ,8aα)]-naphthalene	0.026	0.069	ND
[R-[R*,R*-(E)]]-3,7,11,15-tetramethyl-2-hexadecen-1-ol, acetate	ND	0.010	ND
[R-[R*,R*-(E)]]-3,7,11,15-tetramethyl-2-hexadecene	ND	ND	0.009
1-(1,5-dimethyl-4-hexenyl)-4-methyl-benzene	ND	0.009	0.113
1-(3,4-dimethoxyphenyl)-ethanone	ND	0.002	ND
1-(3-hydroxy-4-methoxyphenyl)-ethanone	0.014	ND	ND
1-(4-methylphenyl)-ethanone	0.011	0.015	0.169
1-(hexahydropyrrolizin-3-ylidene)-3,3-dimethyl-butan-2-one	ND	ND	0.001
1-(phenylmethylene)-1H-indene	ND	ND	0.001
1,1,5-trimethyl-1,2-dihydronaphthalene	ND	ND	0.020
1,1’-oxybis-octane	ND	ND	0.008
1,2,3,4-tetramethyl-benzene	0.019	ND	ND
1,2,3-trimethyl-benzene	ND	0.005	ND
1,2,4a,5,6,8a-hexahydro-4,7-dimethyl-1-(1-methylethyl)-naphthalene	ND	ND	0.132
1,2,4-trimethyl-benzene	0.008	ND	ND
1,2-dihydro-1,1,6-trimethyl-naphthalene	0.002	ND	0.020
1,3,5-triazine	0.004	ND	ND
1,3,5-trimethoxy-benzene	ND	ND	0.005
1,3-bis(1,1-dimethylethyl)-benzene	ND	ND	0.029
1,4-dimethyl-naphthalene	0.002	ND	ND
1,6-dimethyl-4-(1-methylethyl)-naphthalene	ND	0.012	0.099
1,7,7-trimethyl-, (1S)-bicyclo[2.2.1]heptan-2-one	ND	0.044	ND
1,7,7-trimethyl-bicyclo[2.2.1]heptan-2-ol, propanoate	ND	ND	0.012
11-(1-ethylpropyl)-heneicosane	ND	ND	0.011
11-decyl-tetracosane	0.104	0.255	0.019
13-methyltetradecanal	ND	ND	0.020
1-chloro-2-propanol, phosphate (3:1)	ND	ND	0.008
1-decyl-cyclohexene	ND	0.003	ND
1-docosene	ND	0.002	ND
1-dodecanol	ND	ND	0.013
1-eicosanol	ND	0.013	ND
1-ethenyl-1-methyl-2-(1-methylethenyl)-4-(1-methylethylidene)-cyclohexane	ND	ND	0.092
1-ethenyl-1-methyl-2,4-bis(1-methylethenyl)-, [1S-(1α,2β,4β)]-cyclohexane	ND	ND	0.027
1-ethyl-2-propyl-cyclohexane	0.016	0.014	ND
1-hepten-3-one	ND	ND	0.126
1-hexacosene	0.050	0.065	0.002
1-hexadecanol	ND	ND	0.017
1-iodo-docosane	0.008	ND	0.002
1-iodo-dotriacontane	0.067	ND	ND
1-methyl-2-pentyl-cyclohexane	ND	0.009	ND
1-methyl-2-pyrrolidinone	ND	ND	0.001
1-methyl-3-(1-methylethenyl)-benzene	ND	ND	0.007
1-methyl-4-(1-methylethenyl)-1,2-cyclohexanediol	0.022	0.029	ND
1-methyl-4-(1-methylethenyl)-benzene	ND	ND	0.215
1-methyl-4-(1-methylethyl)-1,3-cyclohexadiene/α-terpinene	0.009	0.022	0.695
1-methyl-4-(1-methylethyl)-cyclohexanol	0.010	0.023	ND
1-methyl-4-(1-methylethylidene)-cyclohexene	0.015	0.042	0.037
1-methyl-4-propyl-benzene	0.004	0.010	ND
1-methyl-naphthalene	0.014	0.005	ND
1-nonadecene	ND	0.003	0.003
1-nonanol	ND	0.004	0.089
1-nonen-3-ol	ND	0.015	0.204
1-octadecanol	0.009	ND	ND
1-pentadecene	ND	ND	0.005
1-phenyl-1-propanone	0.015	0.015	0.042
1-tetracosene	ND	ND	0.002
1-tetradecene	ND	ND	0.007
1-tricosene	ND	ND	0.003
1-undecanol	ND	ND	0.005
2-(2-methyl-2-propenyl)-phenol	ND	ND	0.042
2,2′,5,5′-tetrahydro-2,2′-bifuran	ND	ND	0.005
2,2-dihydroxy-1-phenyl-ethanone	ND	ND	0.057
2,3,3,4,7-pentamethyl-2,3-dihydro-benzofuran	ND	ND	0.107
2,3-dehydro-1,8-cineole	0.003	0.027	0.339
2,3-dihydro-benzofuran	0.002	0.001	ND
2,4,6-trimethyl-octane	ND	0.079	ND
2,4-decadienal	ND	0.012	ND
2,4-dihydroxy-3,6-dimethyl-benzoic acid, methyl ester	0.002	0.004	ND
2,4-di-tert-butylphenol	0.039	0.020	0.040
2,6,10,10-tetramethyl-1-oxaspiro[4.5]deca-3,6-diene	ND	ND	0.012
2,6,10,14-tetramethyl-hexadecane	ND	ND	0.009
2,6,10,15-tetramethyl-heptadecane	ND	ND	0.005
2,6,10-trimethyltridecane	ND	ND	0.056
2,6,6-trimethyl-2-cyclohexene-1,4-dione	ND	0.002	0.008
2,6-dimethyl-3,7-octadiene-2,6-diol	ND	0.015	ND
2,6-dimethyl-6-(4-methyl-3-pentenyl)-bicyclo[3.1.1]hept-2-ene	0.007	ND	ND
2,6-dimethyl-octadecane	0.019	ND	ND
2,7,7-trimethyl-bicyclo[3.1.1]hept-2-en-6-one	ND	ND	0.030
2-amino-1,5-dihydro-4H-imidazol-4-one	0.002	ND	ND
2-butenyl-benzene	0.004	ND	ND
2-butyl-1-octanol	0.006	ND	0.003
2-ethyl-1,4-dimethyl-benzene	0.010	0.007	ND
2-ethyl-1-hexanol	ND	ND	0.040
2-fluorobenzoic acid, 2-formyl-4,6-dichlorophenyl ester	ND	ND	0.002
2-fluorobenzoic acid, 4-nitrophenyl ester	0.005	ND	ND
2-hydroxy-3-methyl-1,4-naphthalenedione	ND	0.001	ND
2-hydroxy-benzoic acid, phenylmethyl ester	ND	0.004	0.029
2-methoxy-1-methyl-4-(1-methylethyl)-benzene	0.380	0.656	0.726
2-methoxy-3-(2-propenyl)-phenol	ND	ND	0.078
2-methoxy-4-methyl-1-(1-methylethyl)-benzene	ND	ND	0.872
2-methoxy-4-vinylphenol	ND	ND	0.034
2-methyl-5-(1-methylethyl)-phenol/carvacrol	2.058	1.317	0.958
2-methyl-5-(1-methylethyl)-bicyclo[3.1.0]hex-2-ene	ND	ND	0.006
2-methyl-5-(1-methylethyl)-phenol, acetate/carvacrol acetate	ND	ND	1.231
2-methylbutyl-3-methylbutanoate	ND	ND	0.012
2-methyl-eicosane	ND	ND	0.003
2-methyl-hexadecanal	0.003	ND	ND
2-methyl-octacosane	ND	ND	0.017
2-methyl-octadecane	0.009	ND	ND
2-methyl-pentadecane	ND	ND	0.005
2-methyltetracosane	ND	0.012	0.004
2-phenylethyl-benzoic acid, ester	ND	ND	0.002
2-phenyl-naphthalene	ND	ND	0.002
2-propen-1-ol	ND	ND	0.002
2-propenal	ND	0.003	0.004
2-propenoic acid, anhydride	0.052	ND	ND
2-propenyl-benzene	ND	0.002	ND
2-propyl-furan	0.011	ND	ND
3-(1,1-dimethylethyl)-4-methoxy-phenol	ND	ND	0.032
3,3-diethoxy-1-propyne	ND	ND	0.071
3,4-dihydro-1(2H)-naphthalenone	0.006	0.004	ND
3,4-dimethyl-2,5-furandione	ND	0.005	ND
3,5-diamino-1,2,4-triazole	ND	0.002	ND
3,7,11,15-tetramethyl-2-hexadecen-1-ol	0.040	0.037	0.008
3,7,11,15-tetramethylhexadec-2-en-1-yl acetate	0.015	ND	ND
3,7,11-trimethyl-1-dodecanol	0.005	0.004	0.006
3,7-dimethyl-2,6-octadien-1-ol	ND	0.086	0.224
3,7-dimethyl-undecane	ND	ND	0.003
3,8-dimethyl-undecane	ND	0.003	0.010
3-allyl-6-methoxyphenol	0.002	ND	ND
3-ethyl-4-methyl-1H-pyrrole-2,5-dione	0.008	0.013	ND
3-fluoro-2-propynenitrile	0.011	0.004	0.040
3-hydroxy-benzaldehyde	ND	ND	0.010
3-hydroxypropyl ester oleic acid	ND	0.010	ND
3-methyl-2-cyclohexen-1-one	ND	0.003	ND
3-methyl-6-(1-methylethyl)-2-cyclohexen-1-one	ND	ND	0.195
3-methyl-benzaldehyde	ND	ND	0.066
3-methylhexacosane	ND	0.006	ND
3-methylpentacosane	0.021	0.044	0.007
3-methyl-phenol	ND	ND	0.004
3-octanol	0.035	0.130	0.483
3-pentanol	ND	0.006	ND
4-(2,2,6-trimethyl-7-oxabicyclo[4.1.0]hept-1-yl)-3-buten-2-one	ND	0.011	0.094
4-(2,6,6-trimethyl-1-cyclohexen-1-yl)-3-buten-2-one	ND	0.011	ND
4,11,11-trimethyl-8-methylene-bicyclo[7.2.0]undec-4-ene	ND	ND	0.067
4,5-dimethyl-nonane	ND	ND	0.005
4,6-dimethyl-dodecane	ND	ND	0.006
4,7,7-trimethylbicyclo[4.1.0]hept-3-en-2-one	ND	ND	0.048
4,7-dimethyl-undecane	ND	ND	0.004
4,8,12,16-tetramethylheptadecan-4-olide	0.024	0.036	0.015
4-[(1E)-1,5-dimethyl-1,4-hexadien-1-yl]-1-methyl-cyclohexene	ND	ND	0.086
4-ethenyl-1,2-dimethyl-benzene	ND	0.002	ND
4-hydroxy-3,5,6-trimethyl-4-(3-oxo-1-butenyl)-2-cyclohexen-1-one	ND	0.005	ND
4-hydroxy-3,5-dimethoxy-benzaldehyde	ND	0.001	ND
4-isopropyl-6-methyl-1-methylene-1,2,3,4-tetrahydronaphthalene	0.001	0.003	0.037
4-methoxy-6-(2-propenyl)-1,3-benzodioxole	ND	0.002	0.045
4-methoxybenzoic acid, 2-methoxyethyl ester	ND	ND	0.001
4-methyl-1-(1-methylethyl)-bicyclo[3.1.0]hex-3-en-2-one	ND	ND	0.032
4-methyl-2,4-bis(*p*-hydroxyphenyl)pent-1-ene, 2TMS derivative	0.012	0.035	0.030
4-methyl-4-vinylbutyrolactone	ND	0.004	ND
4-methyl-6-hepten-4-olide	0.002	ND	ND
4-methylene-1-(1-methylethyl)-cyclohexene	ND	ND	0.004
4-tert-octylphenol, TMS derivative	ND	0.011	0.014
5-(1-methylethyl)-bicyclo[3.1.0]hex-3-en-2-one	ND	ND	0.028
5-(4-hexyloxybenzoyloxy)-2-(4-nitrophenyl)pyrimidine	ND	ND	0.001
5-methyl-1,2,3,4-tetrathiane	ND	ND	0.003
5-methyl-2-(1-methylethyl)-cyclohexanol	ND	ND	0.184
5-methyl-2-(1-methylethyl)-phenol, acetate	ND	ND	0.157
5-methyl-2-thiophenecarboxaldehyde	ND	ND	0.004
5β-iodomethyl-1β-isopropenyl-4α,5α-dimethyl-6βbicyclo[4.3.0]nonane	ND	ND	0.003
6,10,14-trimethyl-pentadecan-2-ol	ND	0.003	0.023
6,10-dimethyl-5,9-undecadien-2-ol	ND	0.003	0.038
6,9-heptadecadiene	ND	ND	0.011
6-isopropenyl-4,8a-dimethyl-1,2,3,5,6,7,8,8a-octahydro-naphthalen-2-ol	ND	0.021	ND
6-methyl-3,5-heptadiene-2-one	ND	0.007	0.131
7-hexyl-docosane	0.006	0.006	ND
8-heptadecene	ND	0.008	0.012
9,12,15-octadecatrienal	0.007	ND	ND
9H-fluoren-9-one	ND	ND	0.003
9-methylene-9H-fluorene	ND	0.004	ND
9-methyl-nonadecane	ND	0.006	ND
9-octadecenal	0.156	0.030	0.029
9-octyl-hexacosane	0.056	0.125	ND
Acetic acid, 1,7,7-trimethyl-bicyclo[2.2.1]hept-2-yl ester	0.028	0.093	ND
Aciphyllene	ND	ND	0.017
Ambrosin	ND	0.002	ND
Apocynin	ND	0.017	ND
Aromadendrene oxide-(1)	ND	ND	0.027
Azulene	0.024	ND	ND
Benzaldehyde	0.024	0.024	0.143
Benzeneacetaldehyde	ND	0.004	0.147
Benzothiazole	ND	0.002	ND
Benzyl alcohol	0.013	0.031	0.018
Benzyl benzoate	ND	0.002	0.041
Benzyl nitrile	ND	ND	0.003
Bis(2-ethylhexyl) phthalate	0.017	0.012	0.003
Bornyl acetate	ND	0.019	0.526
Bornyl isovalerate	0.009	ND	ND
Camphor	0.015	ND	0.296
Caprolactam	ND	0.003	ND
Carbonic acid, (1R)-(-)-menthyl tridecyl ester	ND	0.005	ND
Carbonic acid, decyl phenyl ester	ND	0.048	ND
Carbonic acid, nonyl phenyl ester	ND	0.004	ND
Carbonic acid, octadecyl phenyl ester	ND	0.116	ND
Caryophyllene	ND	0.004	0.214
Caryophyllenyl alcohol	0.009	0.019	0.113
*cis*,*cis*,*cis*-7,10,13-hexadecatrienal	ND	0.003	ND
*cis*-3-hexenyl-α-methylbutyrate	ND	ND	0.145
*cis*-dihydrocarvone	0.007	ND	ND
*cis*-linaloloxide	ND	0.005	ND
*cis*-vaccenic acid	10.445	4.269	0.377
Copaene	0.003	0.009	0.162
Coumarin	0.026	0.038	0.012
Dibutyl phthalate	0.052	0.005	0.045
dihydro-3-methylene-5-methyl-2-furanone	ND	0.005	ND
dihydro-5-methyl-2(3H)-furanone	ND	0.011	ND
dihydro-5-pentyl-2(3H)-furanone	ND	ND	0.065
Di-isononyl phthalate	ND	0.069	ND
Dimethyl sulfone	ND	0.005	ND
Diphenyl sulfone	0.001	ND	ND
D-limonene	ND	ND	0.532
Docosane	ND	0.025	0.020
Dodecanal	ND	ND	0.008
Dodecanoic acid	ND	0.014	0.076
Dodecyl acrylate	ND	ND	0.062
Dotriacontanal	ND	0.055	ND
Dotriacontane	ND	0.109	ND
Eicosanal	0.011	0.089	0.007
Eicosane	0.013	0.004	0.008
Endo-borneol	0.298	0.561	0.978
endo-pentanoic acid, 1,7,7-trimethylbicyclo[2.2.1]hept-2-yl ester	ND	ND	0.008
Estragole	ND	ND	0.050
Ethanedioic acid, dimethyl ester	ND	ND	0.001
Ethyl 4-(ethyloxy)-2-oxobut-3-enoate	ND	0.005	ND
Ethylpentamethyl-benzene	ND	ND	0.008
Eugenol	ND	0.004	ND
Fluoranthene	ND	ND	0.007
Fluorene	ND	ND	0.008
Fumaronitrile	ND	0.012	0.015
Geranic acid	ND	0.039	ND
Geraniol	0.468	0.572	1.687
Geranyl acetate	0.092	0.202	0.338
Geranyl formate	0.015	ND	ND
Geranyl isobutyrate	ND	ND	0.119
Geranyl oleate	0.043	ND	ND
Heneicosane	0.018	0.017	0.017
Heptadecane	0.011	0.008	0.024
Hexacosane	ND	ND	0.013
Hexadecanal	ND	0.002	0.015
Hexadecanoic acid, dodecyl ester	ND	ND	0.002
Hexadecanoic acid, methyl ester	ND	0.008	0.030
Hexanoic acid	ND	0.059	ND
Hexatriacontane	0.073	0.710	0.056
Humulene	0.009	0.032	0.331
Hydroxymethyl 2-hydroxy-2-methylpropionate	0.003	ND	ND
Isoaromadendrene epoxide	0.009	0.018	ND
Isophytol	ND	ND	0.036
Isothiazole	ND	0.004	ND
l-Alanine, N-(2-furoyl)-, heptyl ester	ND	ND	0.003
Limonene	0.158	0.318	0.638
Linalool	0.043	0.143	0.346
Linalyl acetate	0.020	0.090	ND
Lup-20(29)-en-3-one	0.053	ND	ND
Lupeol	0.061	0.045	ND
Methyl formate	0.002	ND	ND
Methyl salicylate	ND	ND	0.117
N,N-dimethyl-octanamide	ND	0.002	ND
n-decanoic acid	ND	ND	0.045
Neophytadiene	0.091	0.079	0.014
Neral	ND	ND	0.803
n-hexadecanoic acid	0.413	0.786	0.257
n-nonylcyclohexane	ND	0.002	ND
Nonacos-1-ene	0.008	0.010	ND
Nonanoic acid	ND	0.012	0.068
n-tridecan-1-ol	ND	ND	0.011
O-(2-furoyl)-O’-(pentafluoropropionyl)-1,2-benzenediol	ND	ND	0.020
O,O’-di(4-butylbenzoyl)-1,2-benzenediol	ND	ND	0.135
Octacosane	0.704	0.924	0.012
Octacosanol	ND	0.013	ND
Octadecane	ND	ND	0.022
Octan-2-yl palmitate	ND	ND	0.002
O-dichloroacetyl-O’-(3-methylbut-2-enoyl)-1,2-benzenediol	ND	0.002	ND
Oleic acid	ND	ND	4.312
Oxacycloheptadecan-2-one	ND	ND	0.009
*p*-(1-propenyl)-toluene	0.020	0.031	ND
*p*-(2-methylallyl)-phenol	0.008	ND	ND
*p*-cresol	0.003	0.004	ND
*p*-cumenol	ND	0.004	ND
*p*-cymene	0.130	0.736	0.534
*p*-cymene-2,5-diol	0.104	0.068	0.076
Pentacosanal	ND	0.006	ND
Pentacosane	0.728	0.696	0.044
Pentadecanal	ND	ND	0.033
Pentadecanoic acid	ND	0.011	0.039
Pentamethyl-ethanol	ND	ND	0.004
Pentanoic acid	0.028	ND	ND
Pentyl-benzene	0.005	ND	ND
Phenanthrene	0.002	ND	ND
Phenylethyl alcohol	0.014	0.026	ND
Phloroglucinaldehyde, tris(tert-butyldimethylsilyl) ether	0.004	ND	ND
Phosphorus pentafluoride	ND	ND	0.002
Phthalic acid, cyclobutyl tridecyl ester	ND	ND	0.789
Phthalic acid, hept-4-yl nonyl ester	ND	0.016	ND
Phthalic anhydride	0.003	0.002	ND
Phytol	0.047	ND	0.111
Phytyl decanoate	0.032	0.050	ND
*p*-mentha-1,5-dien-8-ol	0.013	0.030	0.134
Propanoic acid, 2-methyl-, 3-hydroxy-2,2,4-trimethylpentyl ester	ND	0.006	0.016
Propanoic acid, anhydride	ND	0.002	0.047
Propoxy-benzene	ND	0.016	ND
Squalene	0.474	0.813	0.068
Stigmasterol	0.065	0.065	ND
Succinic anhydride	ND	ND	0.004
Sulfur tetrafluoride	0.002	ND	ND
Tetracosanal	0.013	ND	ND
Tetracosane	0.012	0.017	0.007
Tetradecanoic acid	0.010	0.028	0.096
Thymol	ND	ND	0.138
Thymoquinone	0.091	0.117	0.091
*trans*-13-octadecenoic acid	ND	8.888	ND
*trans*-2-(2-pentenyl)furan	ND	ND	0.026
*trans*-2-methyl-5-(1-methylethenyl)-cyclohexanone	ND	0.022	0.195
*trans*-5-methyl-2-(1-methylethyl)-cyclohexanone	ND	ND	0.143
*trans*-geranic acid methyl ester	ND	ND	0.096
*trans*-geranic acid methyl ester	ND	ND	0.096
*trans*-geranylgeraniol	0.046	0.014	ND
*trans*-β-ionone	ND	ND	0.100
Tridecane	ND	ND	0.010
Trifluoroamine oxide	0.002	ND	ND
Undecane	0.018	0.023	0.033
Vanillin	0.011	0.013	ND
Xanthoxylin	0.018	0.029	0.092
α-calacorene	0.005	0.010	0.136
α-corocalene	ND	ND	0.021
α-cubebene	ND	0.003	ND
α-muurolene	0.010	0.023	0.286
α-terpineol	0.241	0.368	0.447
β-amyrin	0.057	0.044	ND
β-amyrone	ND	0.029	ND
β-bisabolene	0.221	0.320	0.563
β-ocimene	0.027	0.062	ND
β-phellandrene	ND	ND	0.538
β-sitosterol	0.133	ND	ND
γ-muurolene	0.012	0.025	0.292
γ-sitostenone	0.067	0.084	ND
γ-terpinene	0.015	0.022	0.394

ND—not detected.

**Table 3:** Antimicrobial activity of wild thyme EO and SFE extracts.

Tested Bacteria		SFE-2 (mg/mL)	SFE-7 (mg/mL)	HD-EO (mg/mL)	CHL (µg/mL) ^1^
*B. spizizeni* ATCC 6633	MIC	0.31 ± 0.00 ^a2^	0.83 ± 0.36 ^b^	0.31 ± 0.00 ^a^	1.95 ± 0.00 ^c^
	MBC	0.31 ± 0.00 ^a^	0.83 ± 0.36 ^b^	0.31 ± 0.00 ^a^	62.5 ± 0.00 ^c^
*E. faecalis* ATCC 29212	MIC	13.33 ± 5.77 ^a^	ND ^3^	1.25 ± 0.00 ^b^	2.60 ± 1.13 ^c^
	MBC	20.00 ± 0.00 ^a^	ND	1.25 ± 0.00 ^b^	ND
*E. faecalis* clinical strain	MIC	5.00 ± 0.00 ^a^	5.00 ± 0.00 ^a^	0.62 ± 0.00 ^b^	1.95 ± 0.00 ^c^
	MBC	10.00 ± 0.00 ^a^	ND	1.25 ± 0.00 ^a^	ND
*S. aureus* ATCC 25923	MIC	<0.02	<0.02	<0.02	<0.98
	MBC	0.31 ± 0.00 ^a^	0.62 ± 0.00 ^b^	0.62 ± 0.00 ^b^	ND
*S. aureus* MRSA clinical stain	MIC	<0.02	<0.02	<0.02	<0.98
	MBC	2.50 ± 0.00 ^a^	2.50 ± 0.00 ^a^	0.62 ± 0.00 ^b^	62.50 ± 00 ^c^
*L. monocytogenes* ATCC 19111	MIC	1.25 ± 0.00 ^a^	1.25 ± 0.00 ^a^	0.16 ± 0.00 ^c^	<0.98
	MBC	5.00 ± 0.00 ^a^	20.00 ± 0.00 ^b^	1.25 ± 0.00 ^c^	ND
*P. mirabilis* ATCC 12453	MIC	6.67 ± 2.69 ^a^	10.0 ± 00.0 ^a^	0.83 ± 0.36 ^c^	1.95 ± 0.00 ^d^
	MBC	10.00 ± 0.00 ^a^	20.00 ± 0.00 ^b^	1.25 ± 0.00 ^c^	ND
*P. hauseri* ATCC 13315	MIC	0.83 ± 0.36 ^a^	2.50 ± 0.00 ^b^	0.16 ± 0.00 ^c^	<0.98
	MBC	2.50 ± 0.00 ^a^	10.00 ± 00.0 ^b^	0.62 ± 0.00 ^c^	500.00 ± 0.00 ^d^
*Ps. aeruginosa* clinical strain	MIC	2.50 ± 0.00 ^a^	5.00 ± 0.00 ^b^	0.62 ± 0.00 ^c^	15.62 ± 0.00 ^d^
	MBC	2.50 ± 0.00 ^a^	5.00 ± 0.00 ^b^	1.25 ± 0.00 ^c^	500.00 ± 0.00 ^d^
*E. coli* ATCC 25922	MIC	ND	ND	1.25 ± 0.00 ^a^	1.95 ± 0.00 ^b^
	MBC	ND	ND	1.25 ± 0.00 ^a^	500.0 ± 0.0 ^b^
*E. coli* H7:O157 ATCC 35150	MIC	ND	ND	1.25 ± 0.00 ^a^	3.91 ± 0.00 ^b^
	MBC	ND	ND	1.25 ± 0.00 ^a^	1000.00 ± 0.00 ^a^
*S.* Enteritidis ATCC 13076	MIC	20.00 ± 0.00 ^a^	ND	2.50 ± 0.00 ^b^	1.95 ± 0.00 ^c^
	MBC	20.00 ± 0.00 ^a^	ND	2.50 ± 0.00 ^b^	ND
*S.* Typhimurium ATCC 14028	MIC	20.00 ± 0.00 ^a^	ND	1.25 ± 0.00 ^b^	1.95 ± 0.00 ^c^
	MBC	20.00 ± 0.00 ^a^	ND	2.50 ± 0.00 ^b^	ND
*S. sonnei* ATCC 29930	MIC	5.00 ± 0.00 ^a^	ND	1.25 ± 0.00 ^b^	0.98 ± 0.00 ^c^
	MBC	ND	ND	2.50 ± 0.00 ^b^	125.00 ± 0.00 ^d^
*Y. enterocolitica* ATCC 27729	MIC	0.83 ± 0.36 ^a^	2.50 ± 0.00 ^b^	0.10 ± 0.04 ^c^	<0.98
	MBC	10.00 ± 0.00 ^a^	ND	0.62 ± 0.00 ^c^	ND
**Tested yeast**		**SFE-2 (mg/mL)**	**SFE-7 (mg/mL)**	**HD-EO (mg/mL)**	**NYS (µg/mL)**
*C. albicans* ATCC 1231	MIC	2.5 ± 0.00 ^a^	ND	1.25 ± 0.00 ^b^	250.00 ± 0.00 ^c^
	MFC	10.00 ± 0.00 ^a^	ND	1.25 ± 0.00 ^b^	250.00 ± 0.00 ^c^

Data are expressed as mean ± standard deviation (n = 3). ^1^ CHL data shown in the table column refer to antibacterial activity of chloramphenicol on the bacterial strains, while NYS refers to antifungal activity of nystatin on *C. albicans*. ^2^ Means marked by different letters in the same row are significantly different at *α* = 0.05 (Tukey’s HSD). ^3^ ND not detected (with the highest tested concentration of samples (20 mg/mL), or antibiotic (1000 µg/L) antimicrobial activity was not detected).

## Data Availability

The data presented in the present study are available in the article and in the Supplementary Materials.

## Supplementary Materials

The following supporting information can be downloaded at: https://www.mdpi.com/article/10.3390/plants13060897/s1, Figure S1: GC chromatograms of (a) SFE-2, (b) SOX-Hex and (c) HD-EO.
