# Supplementary figures and images for: Green Extraction Approach for Isolation of Bioactive Compounds in Wild Thyme (Thymus serpyllum L.) Herbal Dust—Chemical Profile, Antioxidant and Antimicrobial Activity and Comparison with Conventional Techniques
Source: Plants (Basel). 2024 Mar 20;13(6):897. doi: 10.3390/plants13060897 (PMC10975124; doi:10.3390/plants13060897)

## Supplementary material

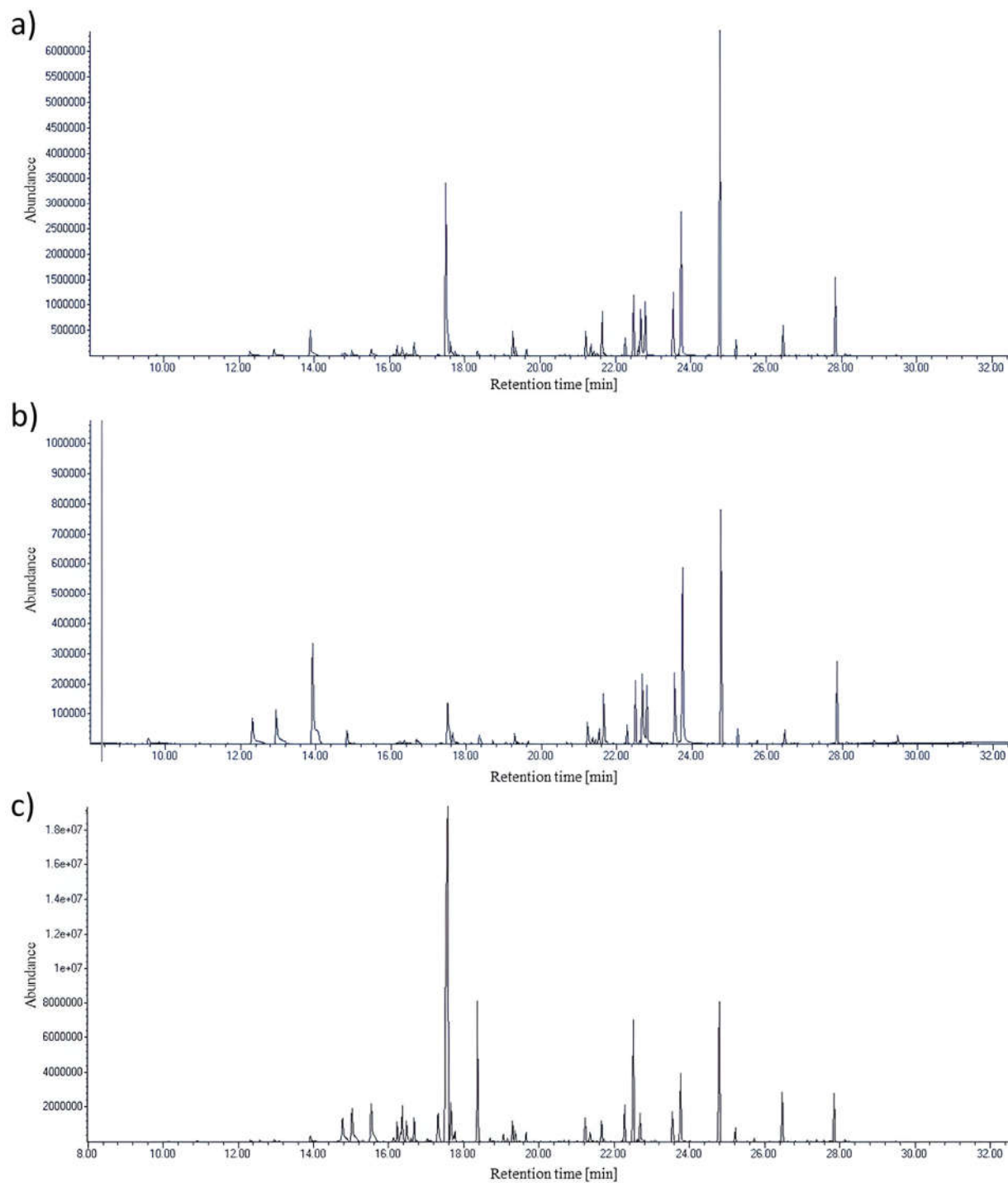

**Figure S1.** GC chromatograms of a) SFE-2, b) SOX-Hex and c) HD-EO

Supplement: Supplementary file 1 [file plants-13-00897-s001.zip › plants-2893768-supplementary.pdf]
